# Supplementary material for: Characterization of self-assembled silver nanoparticle ink based on nanoemulsion method
Source: R Soc Open Sci. 2020 May 27;7(5):200296. doi: 10.1098/rsos.200296 (PMC7277254; doi:10.1098/rsos.200296)

**Supplementary material**

**Article title**

Author 1,^a^ Author 2,^b^ Author 3,^b^ and Author 4^b^

Donghao Hu^a^, Kazuyoshi Ogawa^b^, Mikio Kajiyama^b^ and Toshiharu Enomae^b^

^a^Graduate School of Life and Environmental Sciences, and ^b^Faculty of Life and Environmental Sciences, University of Tsukuba, 1-1-1 Tennodai, Tsukuba, Ibaraki 305-8572, Japan

*^a.^*

*^b.^*

Table S1 Diameter of synthesised AgNPs with different [AgNO_3_] measured with Zetasizer

| [AgNO_3_] (g/mL) | AgNP diameter (nm) | SD (nm) |
| --- | --- | --- |
| 0.10 | 16.9 | 5.3 |
| 0.20 | 18.4 | 4.8 |
| 0.30 | 20.4 | 4.4 |
| 0.50 | 24.2 | 6.5 |

Table S2 Zeta potential of synthesised AgNPs with different [AgNO_3_]

| [AgNO_3_] (g/mL) | Zeta Potential (mV) | SD (mV) | Number of samples measured |
| --- | --- | --- | --- |
| 0.10 | −48.1 | 1.1 | 9 |
| 0.20 | −6.1 | 3.3 | 13 |
| 0.30 | −45.8 | 1.2 | 15 |
| 0.50 | −50.6 | 2.2 | 6 |

Figure S1 DTG curve of solid AgNPs (inner figure is the DTG curve from 40-900 ^o^C).


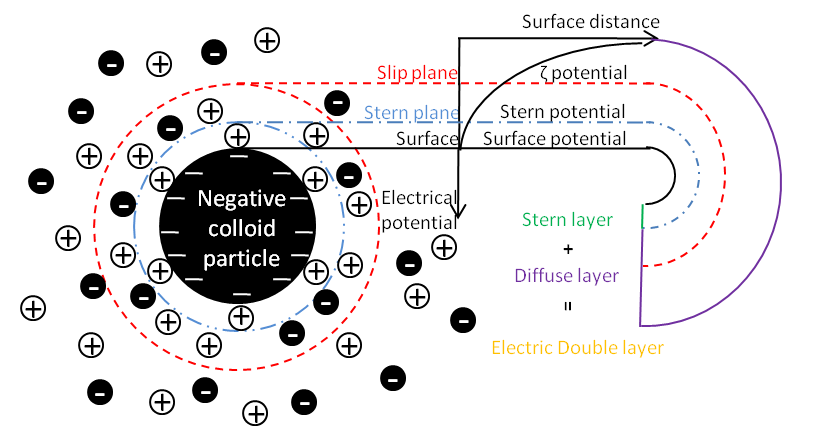


Figure S2 Schematic diagram of (a) electric double layer and (b) *d_H_* measured by DLS and *d_S_* measured by TEM for the AgNPs.


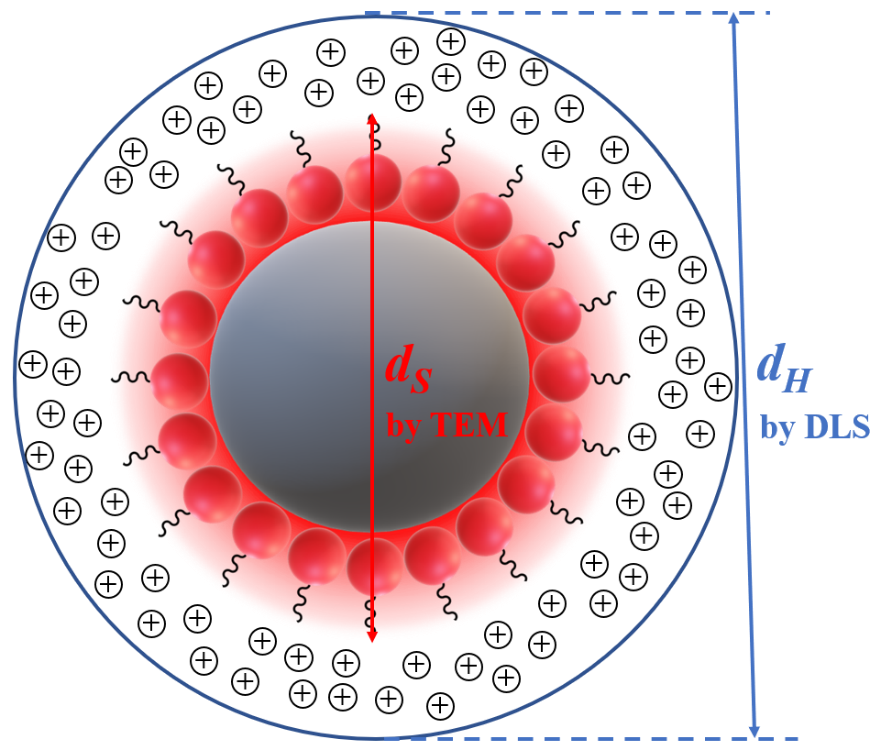

Supplement: Nanoemulsion droplet diameter suggesting stability of the systems [file rsos200296supp1.docx]
